# Cysteine Residues Impact the Stability and Micelle Interaction Dynamics of the Human Mitochondrial β-Barrel Anion Channel hVDAC-2

**DOI:** 10.1371/journal.pone.0092183

**Published:** 2014-03-18

**Authors:** Svetlana Rajkumar Maurya, Radhakrishnan Mahalakshmi

**Affiliations:** Molecular Biophysics Laboratory, Department of Biological Sciences, Indian Institute of Science Education and Research, Bhopal, Madhya Pradesh, India; Indiana University School of Medicine, United States of America

## Abstract

The anti-apoptotic 19-stranded transmembrane human voltage dependent anion channel isoform 2 (hVDAC-2) β-barrel stability is crucial for anion transport in mitochondria. The role of the unusually high number of cysteine residues in this isoform is poorly understood. Using a Cys-less construct of hVDAC-2, we haveinvestigated the contribution of cysteines to channel function, barrel stability and its influence on the strength of protein-micelle interactions. We observe that despite the overall preservation in barrel structure upon cysteine mutation, subtle local variations in the mode of interaction of the barrel with its refolded micellar environment arise, which may manifest itself in the channel activity of both the proteins.Fluorescence measurements of the Trp residues in hVDAC-2 point to possible differences in the association of the barrel with lauryldimethylamine oxide (LDAO) micelles. Upon replacement of cysteines in hVDAC-2, our data suggests greater barrel rigidity by way of intra-protein interactions. This, in turn, lowers the equilibrium barrel thermodynamic parameters in LDAOby perturbingthe stability of the protein-micelle complex. In addition to this, we also find a difference in the cooperativity of unfolding upon increasing the LDAO concentration, implying the importance of micelle concentration and micelle-protein ratios on the stability of this barrel. Our results indicate that the nine cysteine residues of hVDAC-2 are the key in establishing strong(er) barrel interactions with its environment and also impart additional malleability to the barrel scaffold.

## Introduction

Voltage dependent anion channels (VDACs), which are eukaryotic mitochondrial outer membrane proteins [1], conduct metabolites and ionsincluding ATP and NADH [1], [2]between the intermembrane space and cytosol. The three identified isoforms of human VDACscoded by the nuclear DNA are nearly-ubiquitous in all cell types [3], [4]; however, differences in protein expression levels are observed, with hVDAC-1 being the most abundant isoform, whereas hVDAC-2 and hVDAC-3 expressions are generally lowered by one and two orders(s) of magnitude, respectively [4]–[6]. Not surprisingly, hVDAC-1 is also the most extensively characterized, with the structures obtained from both X-ray crystallography and NMR methods pointing to a 19-stranded amphipathic β-barrel structure with an N-terminal helix and barrel closure achieved by parallel hydrogen bonds between strands 1 and 19 [7]–[9].Functionally, VDACs (primarily hVDAC-1) exhibit a conductance of ∼3.5–4.0 nS in 1 M KCl for single channels, between –20 mV and +10 mV, and switch to sub-conductance states above 20–30 mV; additionally, in the closed state, channel selectivity is reversed to cations [2], [5], [10].

In addition to the controlled transport of vital metabolites in the cell, most interestingly, VDACs are deciding elements of mitochondria-mediated apoptosis [4], [11], [12], and have been implicated in several neurodegenerative diseases [5], [13]and cancer [5], [14], [15].Of key significance is the observation that human VDAC isoform 2 (hVDAC-2) may possess antagonistic functions compared to hVDAC-1 [4], [11]. Although hVDAC-2 is expected to share several structural and functional similarities to the other two isoforms, owing to the ∼70% identity between the protein sequences [16], significant differences exist between hVDAC-1 and hVDAC-2 in terms of their added functionality. The presence of all three isoforms is not essential for cell survival; surprisingly, however, unlike isoforms 1 and 3, it was observed that hVDAC-2^−/−^ mice died at the embryonic stage and could not be rescued by the over-expression of VDAC-1 [5], [11], [17]–[19].This led to the serendipitous discovery that hVDAC-2 possesses anti-apoptotic property, likely serves as a specific inhibitor of BAK-dependent mitochondria-mediated apoptosis, and is indispensable for cell survival [5], [11], [17]–[19].

Interestingly, hVDAC-2 is targeted by the anti-tumor agent erastin [20], [21], which triggers a non-apoptotic RAS-RAF-MEK-dependent cell death in tumors upon binding hVDAC-2, through the involvement of reactive oxygen species (ROS) [5], [20]. Most importantly, the metabolite gating of hVDAC-2 is affected by erastin through the generation of ROS [20], [21]. Added to this is the observation that VDAC oxidation causes mitochondrial dysfunction [22]. Remarkably, modeling the hVDAC-2 structure based on isoform-1, positions seven of the nine cysteines in the intermembrane space [23]. Evidence for this non-apoptotic freeradical mediated cytochrome*c* release through hVDACs,resulting in cell death,points to an added functionality facilitated through cysteine residues, which are particularly susceptible to oxidative modifications due to ROS,and are strategically positioned towards the mitochondrial intermembrane space in hVDAC-2 [4], [5], [22].

Accumulating experimental observations place a very important functional role for the cysteines of hVDAC-2. hVDAC-2 and hVDAC-3 could therefore act as cellular buffers to counteract ROS-mediated damage in the intermembrane space [4], as both the proteins are enriched with cysteines (hVDAC-2 has nine and hVDAC-3 has six cysteines). Furthermore, our previous work had indicated an additional biophysical role for cysteines as contributing elements of barrel-lipid interactionsin hVDAC-2, since the mutation of cysteines leads to a less favorable equilibrium free energy of the refolded barrel [24].While the 19-stranded structure of this protein can be readily modeled based on the hVDAC-1 structure, the effect of subtle differences in the protein sequence can only be derived from a systematic investigation of thermodynamic and kinetic factors that, at the molecular level, contribute to hVDAC-2 stability, function and protein-lipid interactions.

Free energy values for the unfolding of transmembrane proteins (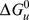) are estimated to be in the range of –2 to –32 kcal mol^−1^ [25]–[30], whereas typical 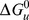
for soluble proteins of comparable sizes are in the –5 to –15 kcal mol^−1^ range [31]. A very intriguing deduction that is increasingly gaining popularity is that membrane proteins are kinetically stabilized, which allows for these systems to exhibit exceptionally long turnaround times and associated high stability within the cell [32], [33].Oligomerization, which is not uncommon in such proteins, further gives rise to a kinetically stabilized mature protein [33]. The downside to kinetic stability is that the protein unfolding process is usually irreversible and estimation of 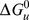
is confounded by hysteresis, wherein the N↔U (native↔unfolded) equilibrium is not attained within reasonable experimental timeframes [27], [33].Nevertheless, it is critical to scrutinizefactors such as the refolding environment, lipid-to-protein ratio, lipid concentration and composition, lipid saturation, chain length and associated curvature, on the stability of biologically relevant proteins such as hVDAC-2.

We have previously compared the differential contribution of thermodynamic and kinetic components towards the overall stability of hVDAC-2 [34]. In this study, we carry out a detailed examination of the dependence of hVDAC-2 barrel (labeled WT) and its Cys-less mutant (labeled C0) on the lauryldimethylamine oxide (LDAO) concentration and its response to chaotropic agents. In stark contrast to the anticipated stability of hVDAC-2, we observe that the barrel is destabilized in high detergent concentrations. Furthermore, we report that mutation of the cysteine residues results in destabilization of protein-LDAO interactions, suggesting that cysteines of hVDAC-2 play a pivotal role as anchoring elements in protein-micelle interactions. We also discuss anunexpected finding that LDAO may serve as a protective agent for hVDAC-2 against urea-mediated barrel denaturation, through a mechanism similar to the osmolyte trimethylamine N-oxide (TMAO).

## Materials and Methods

### Reagents and chemicals

Ultrapure chemicals including LDAO, GdnHCl (guanidine hydrochloride), hexadecane and cholesterol were procured from Sigma-Aldrich Co. LLC. DDM (*n*-dodecyl β-D-maltopyranoside) and DiPhPC (1,2-diphytanoyl 3-phosphocholine) were purchased from Avanti Polar Lipids, Inc. Urea solutions were deionized and used immediately.

### Refolding of hVDAC-2 WT and C0 in LDAO

Wild type hVDAC-2 (WT) and the mutant barrel devoid of cysteines (Cys-less mutant; C0)were cloned in pET-3b, and the protein expressed in*E. coli* BL21(DE3) cells as inclusion bodies was purified using anion exchange,as reported earlier [24]. Protein refolding in 65 mM LDAO micelles prepared in Buffer A (50 mM phosphate buffer pH 7.2 and 100 mM NaCl) by rapid 10-fold dilution from 6 M GdnHCl was carried out at 4°C, to achieve a final protein concentration of 8 μg/μL (250 μM). Aggregated protein was removed by extensive centrifugation and the concentration adjusted to 0.8 μg/μL protein (25 μM) and 65 mM LDAO in Buffer A. A final DTT concentration of 10 mM was maintained in the WT samples. Further dilution of this refolded protein stock was carried out, as given in Table 1, to obtain the LPRs (LDAO-to-protein ratio) described in this study. These samples are labeled in this study as ‘refolded’.

**Table 1:** Summary of the samples and LPRs used in this study.

	LDAO [mM]	Protein	LPR
**Samples prepared by dilution of the refolding stock** a	5	0.06 μg/μL (2 μM)	2600:1
	13	0.16 μg/μL (5 μM)	2600:1
	30	0.16 μg/μL (5 μM)	6000:1
	65	0.16 μg/μL (5 μM)	13000:1
	80	0.16 μg/μL (5 μM)	16000:1
	100	0.16 μg/μL (5 μM)	20000:1
**Directly refolded samples ** ***^b^***	0	0.16 μg/μL (5 μM)	0:1
	5	0.06 μg/μL (2 μM)	2600:1
	5	0.16 μg/μL (5 μM)	1000:1

a Samples labeled ‘refolded’ in the text; *^b^* Samples labeled ‘direct’ in the text.

Samples were also prepared by direct 50-fold dilution of the stock protein in 6 MGdnHCl into either Buffer A or Buffer A + 5 mM LDAO. These samples were labeled as ‘direct’, and were not centrifuged prior to use, in order to retain the aggregated protein (if any). hVDAC-2 WT and C0 were also refolded in DDM using the same procedure described for LDAO, with the exception that 1% DDM was used instead of LDAO, in Buffer A. A 5-fold dilution of the refolding reaction was carried out to obtain a final protein concentration of 5 μM in 0.2% DDM (in Buffer A).

### Channel conductance measurements on planar bilayers

Activity of refolded hVDAC-2 WT and C0 was measured using the black lipid membrane system (Warner Instruments). Planar lipid membranes composed of DiPhPC and 0.1 % cholesterol were painted on a 200 μm aperture in a polysulfone chamber, using the Mueller-Rudin method [35]. The *cis* and *trans* chambers were filled with 10 mM HEPES pH 7.4, 5 mM CaCl_2_, and 1 M KCl. Freshly refolded hVDAC-2 in 65 mM LDAO was incubated with 1% Triton X-100 + 0.1 % cholesterol for 30 min on ice [8], [36], and 0.5–1 μL aliquots were added to the *cis* side at a holding voltage of +10 mV to achieve channel insertion. A triangular voltage ramp [8], [36] from +60 mV to –60 mV at 3 mV/s was applied, and data was collected at 10 kHz with a sampling frequency of 1 kHz [42]; data reduction was carried out by a factor of 10 during analysis.

### Circular dichroism (CD) spectropolarimetry

CD wavelength scans were recorded at 4°C using a scan rate of 100 nm/min, 1 s response time, 1 nm bandwidth and an optical path length of 0.1 or 0.2 cm. An average of three scans was acquired and buffer contribution was subtracted before conversion to molar ellipticity using reported methods [37]. No significant difference was observed between spectra recorded at 4°C and 25°C (not shown).

### Cross-linking,protease digestionand mass spectrometry

Refolded’ WT and C0 as well as the ‘direct’ samples (controls) in various LDAO concentrations were subjected to 2% formaldehyde (cross-linking experiments) or 20 ng/μL trypsin treatment (protease digestion experiments) for 10 min, at 25°C. All reactions were quenched using SDS-PAGE gel loading dye (additionallycontaining 5 mM PMSF for the trypsinization experiments) and analyzed on 12% Laemmli gels. Densitometry was carried out by ImageJ [38], andquantification of cross-linked protein was achieved using the formula:
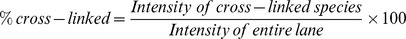



Mass spectrometric analysis was carried out on a MALDI-ToF/ToF mass spectrometer using reported protocols [24].

### Fluorescence anisotropy, quenching and lifetime measurements

Trp fluorescence anisotropy was measured at 25°C using λ_ex_  =  295 nm and λ_em_  =  340 nm or 360 nm for folded and unfolded proteins, respectively, using reported protocols [39]. Acrylamide quenching was carried out at 25°C as described earlier [24], without the inner filter correction. Time-correlated single photon counting was used to obtain fluorescence lifetime measurements. Samples were excited at 292 nm and the data was collected at either 340 nm or 360 nm. Instrument response function (IRF) measured using 0.01% LUDOX AS-40 solution was typically ∼850 ps. Fitting of all the decay curves was carried out using atriple exponential function (Figure S1 in File S1), as described previously [24].Bimolecular quenching constant (*k*_q_) was calculated as described previously [40].

### Chemical denaturation using steady state fluorescence experiments

Equilibrium unfolding using urea or GdnHClwas monitored using total Trp fluorescence (hVDAC-2 has four Trp residues). Refolded sample stocks were 5-fold diluted in the denaturant gradient, and incubated for 1 h at 25°C, to attain equilibrium. Emission spectra were acquired between 320 – 400 nm, using a λ_ex_  =  295 nm, as reported earlier [39]. Since hysteresis was observed in many of the conditions used, only the apparent free energy of unfolding (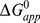), at 0 M denaturant [29], was calculated from the unfolded fractions, using the equation [41]:
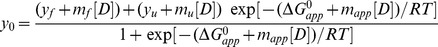



Here, *y_0_* is the observed fluorescence intensity at a particular denaturant concentration *D*, *y_f_* and *m_f_* are the intercept and slope of the native protein baseline, *y_u_* and *m_u_* are the intercept and slope of the unfolded protein baseline, *m_app_* represents the apparent cooperativity of the unfolding reaction. R is the gas constant and T  =  298 K. The midpoint of chemical denaturation (*C_m_*)was calculated using the equation *C_m_*  =  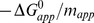. Due to the absence of defined pre- and post-transition baselines in 80 and 100 mM LDAO samples, thermodynamic parameters were derived by linear extrapolation [42].

## Results

### Effect of the nine cysteine residues of hVDAC-2 on barrel activity

**Figure 1:**
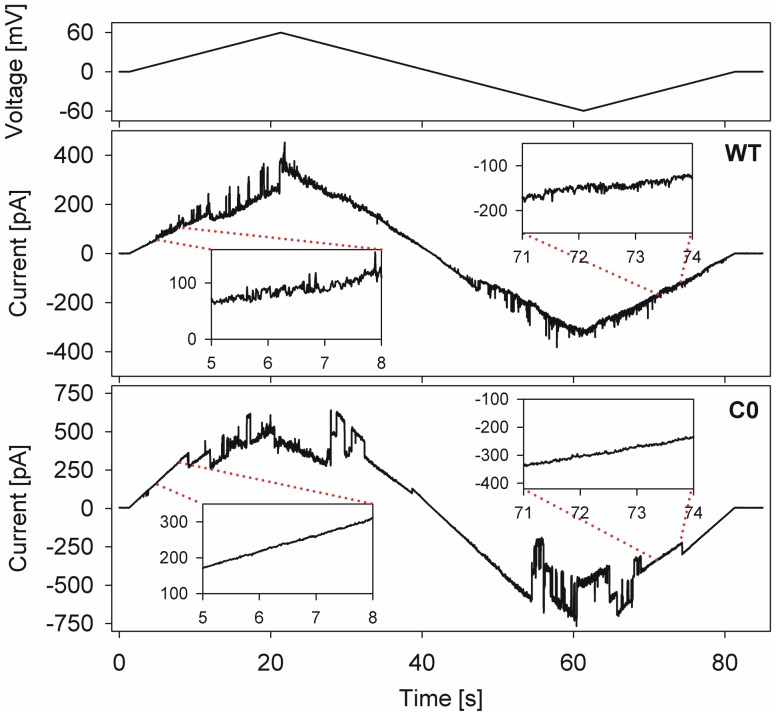
Channel conductance measurements of hVDAC-2 WT and C0 against a voltage ramp. Representative results of voltage ramp experiments that compare the channel conductance of full-length hVDAC-2 WT and C0 protein. The upper panel shows the applied voltage ramp, which ranges from +60 mV to –60 mV over a period of 84 s. The response of hVDAC-2 WT (middle panel) and C0 (lower panel) are shown. The steep slope of the conductance at lower voltages corresponds to the open state of the channel while the shallower slopes at higher voltages correspond to the closed state of the hVDAC-2 channel. In both the cases presented here, the membrane had ∼3–4 active channels.Representative segments of the traces are expanded in the insets to highlight the difference in noise levels of both proteins.

In order to address the role of cysteines in hVDAC-2, we generated a Cys-less mutant (C0), by replacing the nine cysteines with the corresponding amino acid present in hVDAC-1 or hVDAC-3 (Figure S2 in File S1).We assessed barrel behavior by monitoringthe response of either LDAO-refolded hVDAC-2 WT or C0 to a voltage ramp between -60 mV to +60 mV after it was incorporated in DiPhPC bilayers (Figure 1, and Figure S3 in File S1). Overall, both proteins exhibit channel activity typical for VDACs, with conductance being obtained below ∼30 mV, and the barrel attains a closed state at higher voltages (Figure 1, and Figure S3 in File S1).Channel conductance was ∼3.5 nS in 1 M KCl at lower voltages, although frequently, channels of lower conductance were also incorporated (data not shown). Previous studies on hVDAC-2 cloned in yeast [3] and VDAC-2 directlypurified from enriched fractions of bovine spermatozoa [43] have shown two different populations of hVDAC-2 being incorporated in the lipid bilayers,which have ∼4.0 nS and ∼2.5–3.0 nS conductance, along with ∼1.5 nS short-lived conductance states. Thegreater incidence of sub-conductance states in hVDAC-2, at holding voltages that result in open channels in hVDAC-1, which has been reported earlier [3], [43], is also observed in our recordings of hVDAC-2. A careful observation of the data, however, suggests that subtle differences exist in the observed conductance (inset of Figure 1, and inset of Figure S3 in File S1). C0 showsmore pronounced transitions between the open and closed states, especially at higher voltages (Figure 1, and Figure S3 in File S1). Moreover, we obtained noisier data for hVDAC-2 WT, which was seemingly independent of the protein batch. It is therefore likely that both the hVDAC-2 WT and C0 we have refolded in LDAO micelles and incorporated in DiPhPC bilayers bears some semblance to the natively purified VDAC-2 [3], [43] and artificially reconstituted VDAC-1 from micellar systems [3], [9], [22], [44], [45], respectively. We have observed earlier that mutation of cysteine residues results in altered biophysicalproperties of the barrel [24]. Could the incidence of alternate conductance statesreported in the literature [3], the noisier data we obtain in the case of WT hVDAC-2be attributed to the cysteines (or absence thereof), which gives rise to local variations in the barrel scaffold and associated structural and functional properties of hVDAC-2? To probe this aspect further, we examined behavioral features of both WT and C0 barrels in LDAO.

### hVDAC-2 barrel formation depends on LDAO-to-protein ratio but oligomerization is not

**Figure 2:**
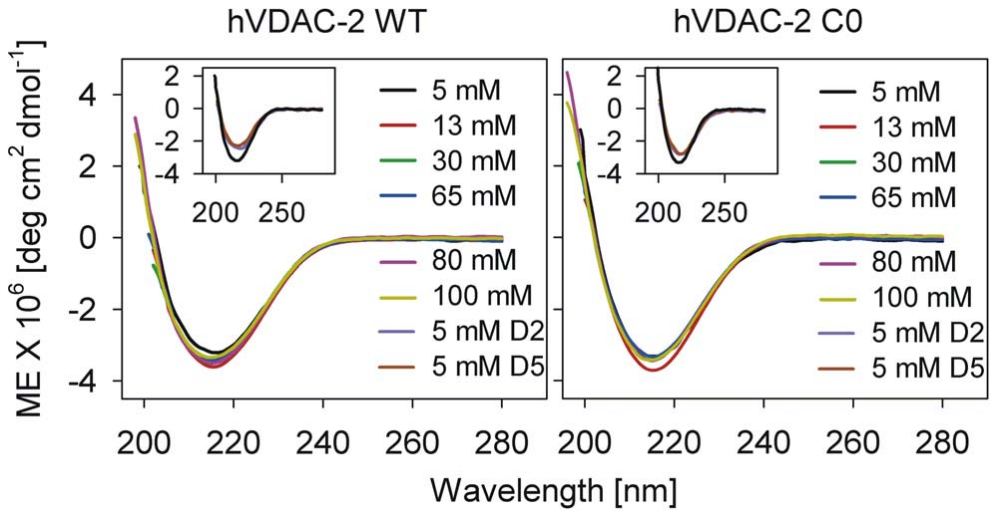
Representative far-UV circular dichroism profiles of refolded hVDAC-2 WT and C0 with increasing LDAOconcentrations. Both WT (left) and C0 (right) display CD spectra corresponding to an extended conformation, with a negative maximum at ∼215 nm. Both proteins exhibit comparable secondary structure content, despite a 20-fold change in the LDAO concentration from 5 mM to 100 mM. The inset shows the CD spectra of control samples prepared by ‘direct’ refolding in LPRs of 2600∶1 (5 mM D2) and 1000∶1 (5 mM D5), and are compared with ‘refolded’ protein in 5 mM LDAO, also having an LPR of 2600:1. Molar ellipticity (ME) values are lower for the control samples, suggesting the importance of absolute LDAO concentrations to mediate optimal refolding.

We first probed the effect of LPR on the structural features of both barrels by determining the secondary structure content of WT and C0 using far-UV CD.Wavelength scans of both refolded proteins reveal no significant change in β-sheet content upon changing the LPR from 2600:1 to 20000:1 (Figure 2; ‘refolded’ samples). However, when we assessed the ability of either protein to refold directly in low LPRs (1000∶1–2600∶1) by lowering the absolute LDAO concentration in the refolding reaction, we observe an ∼1.5-fold loss in secondary structure (Figure 2 inset; ‘direct’ samples), implying the importance of absolute LDAO concentration during the barrel refolding process. It is likely that hVDAC-2 refolding requires the presence of sufficient micellar forms (∼0.06 mM micelles in 5 mM ‘direct’ *vs* ∼0.85 mM micelles in 65 mM ‘refolded’ sample preparations, considering a critical micelle concentration for LDAO of ∼0.14 mM in NaCl [46]) to attain the refolded state *in vitro*, as observed previously for *E. coli* OmpA [47].

We next probed the effect of cysteine replacement on barrel oligomerization. While mass spectrometric analysis has established that disulfides are largely absent in refolded hVDAC-2, disulfide-mediated oligomerization may be induced as a consequence of changes in LPR. However, we observe comparable oligomerization (which together constitute ∼40% of the total protein; ∼60% stays monomeric) in both WT and C0 barrels in all LPRs, in formaldehyde-mediated cross-linking experiments(Figure S4 in File S1).However, we do not detect defined dimeric and trimeric forms observed previously for hVDAC-1 [48] and rat liver mitochondrial VDAC [49], which could be attributed either to the tendency of hVDAC-2 to form multimeric structures, or to non-specific cross linking by formaldehyde.The presence of a two-fold excess DTT (the default concentration is 2 mM DTT) in WT samples showed an additional increase in the cross-linked species (∼55% oligomers), that can arise due to the increase in cross-linking efficiency upon membrane association of Cys-containing proteins, as observed earlier [50]. The oligomerization was more profound when other cross-linking agents such as glutaraldehyde or EGS were used in place of formaldehyde (data not shown). Also, dynamic light scattering measurements suggested the existence of only the monomeric form in high LPRs (data not shown), which is inconsistent with our cross-linking experiments (Figure S4 in File S1)., It is likely that oligomerization of hVDAC-2 refolded in LDAO micelles is alargely diffusion-driven non-specific association event that is not influenced by LPR.Diversity in the observed multimeric species, however, precludes accurate mapping of the interaction interface of hVDAC-2.

**Figure 3:**
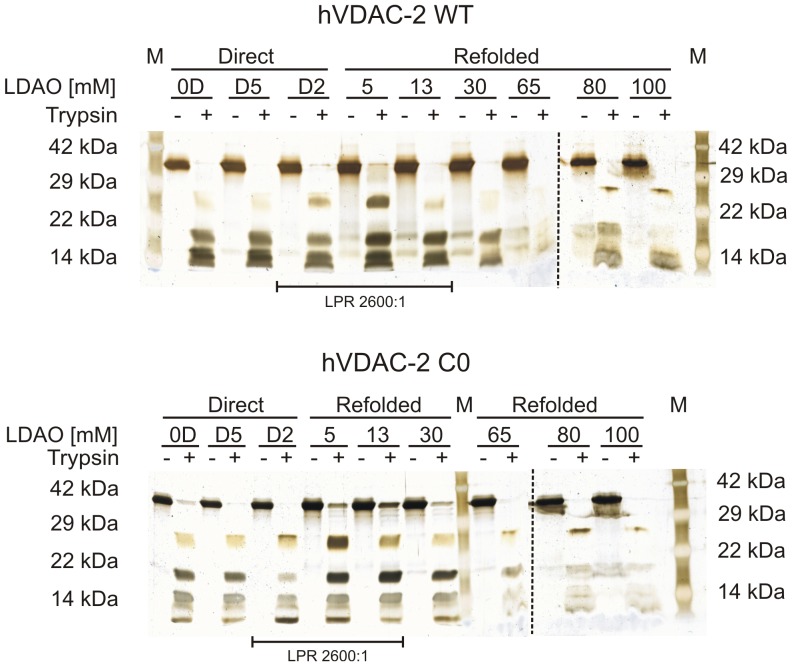
Monitoring the susceptibility of refolded hVDAC-2 to trypsinization with increasing LDAO. Representative silver stained SDS-PAGE gels of refolded WT (top) and C0 (bottom) subjected to a 10 min exposure to trypsin (+) and arrested by the addition of 5 mM PMSF are compared with undigested samples (–). The LDAO concentrations in each sample are indicated above each lane and the refolding method employed is also indicated (samples 0D, 2D and 5D are directly refolded and labeled ‘Direct’; those labeled ‘Refolded’ were generated by dilution of the refolded stock; see Materials and Methods for details of sample preparation). Note that D2, 5 and 13 samples possess a similar LPR of 2600:1. The observed distortion in protein migration, particularly in high LPR, is due to the presence of excess LDAO in the sample, which interferes in proper gel running. Relevant molecular weight standards (M) are indicated on either side of each gel. Dashed lines are used to separate different gels that are presented together.

To probe the effect of LPR and cysteinemutation on hVDAC-2 barrel structure, we examined the susceptibility of the refolded protein to proteases such as trypsin. Interestingly, with the exception of the 5 mM ‘refolded’ sample, all other protein preparations demonstrated variable susceptibility to trypsinization (Figure 3). A persistent ∼32 kDa band corresponding to the intact proteinis observed in most of the ‘refolded’ samples exposed to trypsinization, especially at lower LDAO concentrations (5 – 30 mM LDAO).Additionally, the observation of ∼17 kDa, ∼20 kDa and ∼26 kDa bands suggest that the R/K-rich centers distributed between strands 7–11 are readilysusceptible to trypsin. However, the lack of a defined demarcation in protease susceptibility of hVDAC-2 WT and C0, between the various LPRs, prevents us from drawing definitive conclusions from these experiments. A peculiar observation is the similarity in the digestion pattern of aggregated and LDAO-refolded hVDAC-2 WT and C0 samples (lanes 0D, D2 and D5 in Figure 3), suggesting similar trypsin-susceptible sites in these samples. A previous study has suggested the likely persistence of residual structure for hVDAC-1 in the absence of any lipid [44]. We therefore compared the far-UV CD spectra of both WT and C0 in buffer (Figure S5 in File S1), which indicates the presence of substantial β-sheet content in hVDAC-2 that wasprepared by dilution from urea. It is likely that theresults of our trypsinization experiments arise due to similarly exposed protease sites in aggregated and refolded hVDAC-2 preparations. We examined the peptide mass fingerprints of the LPR 0∶1 and 2600∶1 samples for similarities in the tryptic fragments (Figure S6 in File S1). Poor ionization of the refolded samples currently prevents us from deriving meaningful correlation between the peptide fragments of the aggregated and refolded proteins in both WT and C0.

### Fluorescence measurements indicate subtle variations in the local Trp environment upon cysteine mutation

**Figure 4:**
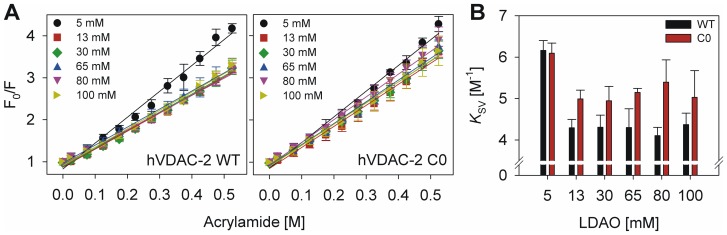
Probing surface accessibility of hVDAC-2 Trp residues using fluorescence quenching measurements. (A) Acrylamide quenching experiments of Trp fluorescence in refolded hVDAC-2 WT (left panel) and C0 (right panel) in different LDAO concentrations, fit to a linear function (fits are shown as solid lines). Protein refolded in 5 mM LDAO exhibits greater quenching with increasing acrylamide, which is a reflection of solvent-accessible Trp residues in this. Notably, the slopes of quenching measurements in C0 are consistently greater, even in higher LDAO, suggesting that the tryptophans remain acrylamide accessible in this protein. (B) *K*_SV_ of hVDAC2 WT and C0 obtained from acrylamide quenching of Trp fluorescence at 25°C, are compared in different LDAO concentrations. The error bars in all graphs indicate standard deviations from three independent experiments.

At the molecular level, we found it intriguing to assess the effect of cysteine mutations on barrel structure, using fluorescence measurements. hVDAC-2 has four intrinsic tryptophan residues, that are positioned at the lipid-protein interface (Figure S7 in File S1). The observed anisotropy and lifetime values for both barrels in various LPRs are summarized in Table 2. Interestingly, while the anisotropy values for both proteins are comparable under most of the conditions for both WT and C0, the average fluorescence lifetimes(<τ>) are different, with the WT displaying values between 2.6 ns –2.8 ns, whereas the <τ> for C0 is ∼3.2 ns.This suggests that Trp residues of WT either have neighboring quencher residues (cysteine residues) or those of C0 are likely to possess less conformational flexibility. To check the Trp accessibility further, we carried out acrylamide quenching measurements, which provided us with marginally elevated Stern-Volmer constants (*K*_SV_) for C0(Figure 4). This data indicates the presence of acrylamide-accessible indole residues in C0 [40]. Since the accessibility of the tryptophan depends on its solvent exposure and relates to the observed fluorophore lifetimes [40],wederived the exposure of the tryptophans in both barrels using the *k*_q_ values. We obtained values of ∼1.5×10^9^ M^−1^ s^−1^ for both WT and C0 (Table 3) in the various LDAO concentrations (13 mM – 100 mM), suggesting that the Trp residues in both proteins share a similar degree of solvent exposure.This*k*_q_ value for acrylamide quenching indicates moderate level of exposure of the indole rings [51], which is expected for interfacial Trp residues of transmembrane proteins.

**Table 2:** Summary of Trp fluorescence lifetime and anisotropy values of WT and C0 refolded in various LPRs.

LDAO concen-tration (LPR)	WT						C0					
	Folded protein			Denatured using 6 M GdnHCl a			Folded protein			Denatured using 6 M GdnHCl a		
	r	<τ>*^b^*	?^2 *c*^	r	<τ>*^b^*	?^2 *c*^	r	<τ>*^b^*	?^2 *c*^	r	<τ>*^b^*	?^2 *c*^
0 mM(0∶1) *^d^*	0.168	ND*^f^*	ND*^f^*	0.068	1.57	1.10	0.156	ND*^f^*	ND*^f^*	0.059	1.72	1.12
5 mM D2 (2600∶1) *^d^*	0.139	2.21	1.05	0.062	1.71	1.07	0.138	2.65	1.13	0.054	1.70	1.09
5 mM RF (2600∶1) *^e^*	0.131	2.63	1.07	0.052	1.59	1.03	0.129	3.11	1.05	0.051	1.63	0.98
13 mM RF (2600∶1) *^e^*	0.134	2.81	1.03	0.061	1.73	1.09	0.131	3.22	1.04	0.060	1.77	1.05
30 mM RF (6000∶1) *^e^*	0.132	2.72	1.06	0.063	1.78	1.14	0.130	3.24	1.02	0.061	1.83	1.14
65 mM RF (13000∶1) *^e^*	0.132	2.82	1.05	0.066	1.92	1.06	0.130	3.21	1.06	0.061	2.04	1.08
80 mM RF (16000∶1) *^e^*	0.133	2.81	1.07	0.067	1.97	1.13	0.129	3.21	1.07	0.062	2.08	1.06
100 mM RF (20000∶1) *^e^*	0.133	2.80	1.06	0.069	1.93	1.12	0.130	3.22	1.00	0.063	2.14	1.01

a Changes observed in Trp lifetime and anisotropy of the denatured samples can be explained by taking into account the change in viscosity of the sample; *^b^* Average tryptophan lifetimes obtained from fits to a three exponential function. The values provided are in ns; *^c^* Goodness of fit for the fluorescence lifetime measurements; *^d^* Samples have trace amounts of GdnHCl due to direct (D) refolding; 5 mM D2 corresponds to 2 μM protein directly refolded in 5 mM LDAO; *^e^* Samples have been diluted from the refolded stock (RF) solution; *^f^* ND – Not determined, due to scattering by aggregated protein.

**Table 3:** Bimolecular quenching constants for refolded hVDAC-2 WT and C0 in various LDAO concentrations.

LDAO (LPR)^a^	hVDAC-2 WT (X 10^9^ M^−1^ s^−1^)	hVDAC-2 C0(X 10^9^ M^−1^ s^−1^)
5 mM RF (2600∶1)	2.34	1.96
13 mM RF (2600∶1)	1.53	1.55
30 mM RF (6000∶1)	1.58	1.53
65 mM RF (13000∶1)	1.52	1.60
80 mM RF (16000∶1)	1.46	1.68
100 mM RF (20000∶1)	1.56	1.56

a Samples labeled ‘refolded’ in the text are indicated as RF.

The calculated *K*_SV_ values for both WT and C0 are independent of LPR (Figure 4), which suggeststhat the barrel conformation remains largely unchanged when the LDAO concentration is varied after protein folding. This is in line with the far-UV CD measurements shown in Figure 2. Additionally, the refolded sample shows higher *k*_q_ values only in the case 5 mM LDAO,despite having a similar LPR (2600∶1) as the 13 mM sample, suggesting the presence of highly exposed tryptophansat this LDAO concentration. Also, the lifetime measurements of the ‘direct’ samples refolded in 5 mM LDAO provides us with values of 2.21 ns and 2.65 ns for WT and C0, respectively, whereas the corresponding values in samples that were diluted after refolding (in the same LDAO concentration of 5 mM) are 2.63 ns and 3.11 ns, respectively, for WT and C0 (Table 2). Our lifetime data indicates the presence of exposed indole rings in 5 mM ‘direct’ samples that can exhibit greater degrees of rotational motion at the side chain torsion angles χ_1_ and χ_2_,thereby lowering their lifetimes. Our fluorescence measurements indicate that in addition to the cysteine residues, the absolute LDAO concentration is also important to maintain the overall barrel structure and rigidity.

### LDAO hinders urea-mediated hVDAC-2 unfolding

**Figure 5:**
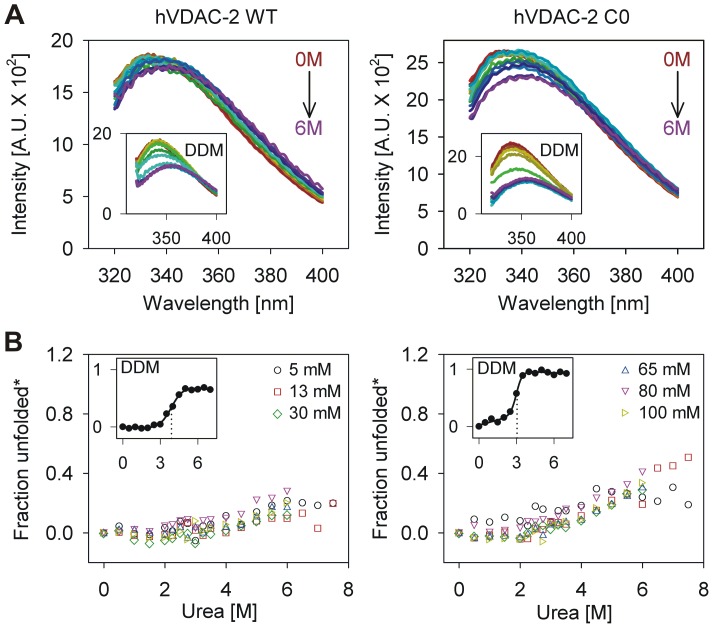
Equilibrium unfolding studies of refolded hVDAC-2 in urea. (A) Representative Trp fluorescence wavelength scans of hVDAC-2 WT (left) and C0 (right) refolded in 13 mM LDAO and 0.2 % DDM (shown as insets) after 1 h incubation at 25°C in a urea gradient. Note the heavy overlap in the emission spectra in the case of LDAO refolded samples, with only a marginal red shift of the λ_em-max_ (∼3–4 nm), suggesting that hVDAC-2 does not undergo significant protein unfolding in this condition. However, samples in DDM (insets) display a > 15 nm red-shifted λ_em-max_ accompanied by a significant reduction in the emission intensity in higher urea concentrations, indicative of substantial barrel unfolding. (B) Plot of unfolded fractions for hVDAC-2 WT (left) and C0 (right) calculated using 340 nm intensity values, in the various LDAO concentrations used in this study, after 1 h incubation at 25°C in urea. The unfolded fractions were calculated by using values for the unfolded protein from GdnHCl, and are hence indicated by (*). LDAO underwent precipitation above 6 M urea in concentrations >13 mM; hence these values are not included in the final analysis. This precipitation appears to be a property of LDAO when both the detergent and urea are in high concentrations, as it was also observed in samples without the protein. Data points shown are the mean of three datasets. Error bars are omitted for clarity. The insets show the corresponding unfolded fractions calculated for urea denatured hVDAC-2 WT and C0 refolded in 0.2% DDM, obtained under similar experimental conditions and analyzed as described for LDAO. Solid lines represent fits to a two-state equation, and the dotted line highlights the calculated *C_m_*. Note that the extent of unfolding of C0 is greater in LDAO and DDM (compare fraction unfolded* values of WT and C0 in 4 – 8 M urea). Furthermore, the *C_m_* of C0 is lower than WT in DDM (3.9 M for WT and 3.0 M for C0).

Potential alterations in the barrel-LDAO interaction interface can be readily assessed using destabilizing agents such as chemical denaturants. Thermodynamic stability estimated from chemical denaturation experiments also provides anexcellent comparison between WT and C0 and the effect of cysteine replacement on barrel energetics. Urea has been one of the denaturants of choice for equilibrium unfolding measurements of transmembrane β-barrels extensively examined so far, such as OmpA [29], [52], PagP [26] and GalP [53]. Using the intrinsic fluorescence of the four Trp residues of hVDAC-2, we acquired data for barrel unfolding as a function of time.Surprisingly, we find that both hVDAC-2 WT and C0 do not undergo complete unfolding even after 24 h incubation in 7.5 M urea, at the lowest LDAO concentrations used in this study (Figure 5).This is unexpected, particularly when we consider that the thermodynamic stability of hVDAC-2 in LDAO micelles is considerably lower (1.8 – 4.5 kcal/mol [24] than soluble proteins of comparable molecular weights (8– 25 kcal/mol [54]–[57]).While both urea and GdnHCl have been employed in equilibrium thermodynamics studies, a few bacterial transmembrane β-barrels are known to exhibit a differential response to urea as a denaturant. For instance, *E. coli* PagP transforms from a folded to an adsorbed state with increase in urea [28], whereas the structurally related 8-stranded barrel, OmpA, displays hysteresis [52]. However, both studies were carried out in the more stablevesicle systems, whereas LDAO forms micellar structures.

Does the negligible unfolding of hVDAC-2 therefore represent an LDAO-adsorbed state? To examine the source of this “stability” of refolded hVDAC-2 in urea, we examined urea-mediated unfolding of both WT and C0 in another 12-C non-ionic detergent DDM, which possesses physico-chemical properties that are similar to LDAO.For example, the aggregation number (LDAO: 76; DDM: 78-149) ( [58] and references therein) and critical micelle concentrationin the presence of salt (LDAO: ∼0.14 mM [46], [58]DDM: ∼0.2 mM [58]) are comparable for both detergents. The major difference lies in the head group, which in turn affects the micelle size (LDAO: ∼21 kDa; DDM: ∼72 kDa) [59]. Representative fluorescence emission spectra obtained in DDM are compared with the corresponding LDAO samples in Figure 5A. We obtained near-complete barrel unfolding in DDM within 1 h of equilibration, along with defined pre- and post-transition baselines (Figure 5B inset). This suggests that the barrel stability to urea we observe in LDAO is likely to be a property conferred on the barrel by the dimethylamine oxide headgroup of this detergent.

Interestingly, we observe that although the unfolding process is incomplete in urea, C0 undergoes a greater loss in Trp fluorescence when compared to WT. For instance, in LDAO, WT undergoes up to ∼20% unfolding in 7.5 M urea, whereas C0 achieves similar unfolding levels in ∼4.5 M urea. Similarly, in DDM, although the unfolding process is cooperative in both proteins, C0 undergoes complete unfolding, whereas WT does not. This could possibly reflect the lowered affinity of C0 to LDAO and DDM, which is in line with our previous observations, as well as corroborates our results from acrylamide quenching experiments (Figure 4). Therefore, do cysteine residues increase the barrel affinity to its lipid or detergent environment? To address this further, we examined the equilibrium unfolding of both barrels in GdnHCl.

### Unfolding process is less cooperative in high LDAO,with WT more denaturant-tolerant than C0

**Figure 6:**
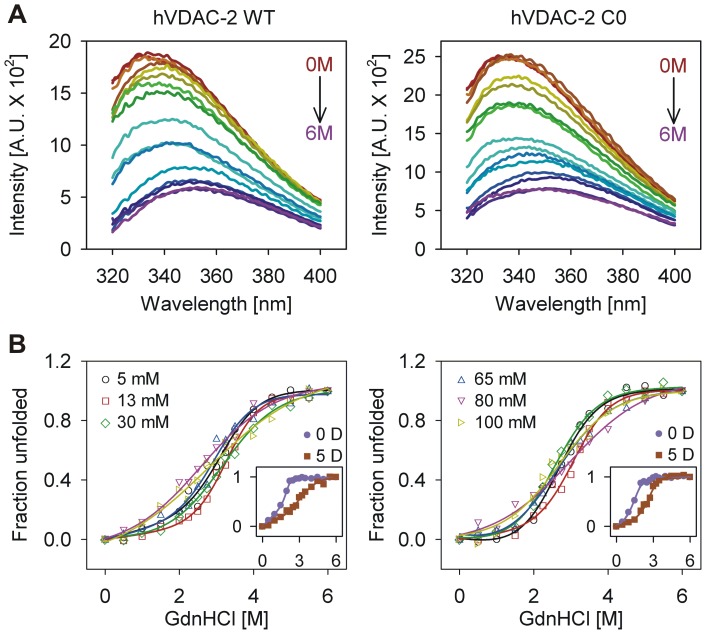
Equilibrium unfolding studies of refolded hVDAC-2 using GdnHCl. (A) Representative Trp fluorescence wavelength scans of refolded hVDAC-2 WT (left) and C0 (right) in 13 mM LDAO in a GdnHCl gradient, recorded after 1 h incubation at 25°C. Note the decrease in intensity as well as the red shifted λ_em-max_ in GdnHCl, indicating near-complete protein unfolding. (B) Refolded hVDAC-2 WT (left) and C0 (right) in different LDAO concentrations were subjected to an increasing GdnHCl gradient at 25°C. The plots represent unfolded fractions derived from the change in Trp fluorescence intensity at 340 nm, as the protein unfolds. Solid lines denote fits to a two-state equation, except in the case of 80 mM and 100 mM experiments, wherein a sigmoidal fit was used to illustrate the trend in the dataset. Also shown, as insets, are the unfolding curves obtained for aggregated protein (protein in buffer; 0 D) and hVDAC-2 refolded directly in 5 mM LDAO (5 D; LPR of 1000:1). Note the shift in *C_m_* for the former and/or loss in cooperativity in the latter. The color scheme used in Figure 5 for the various samples is also retained here. Legends are distributed in the left and right panel. Data points shown here are the mean of three datasets and error bars are omitted for clarity.

**Figure 7:**
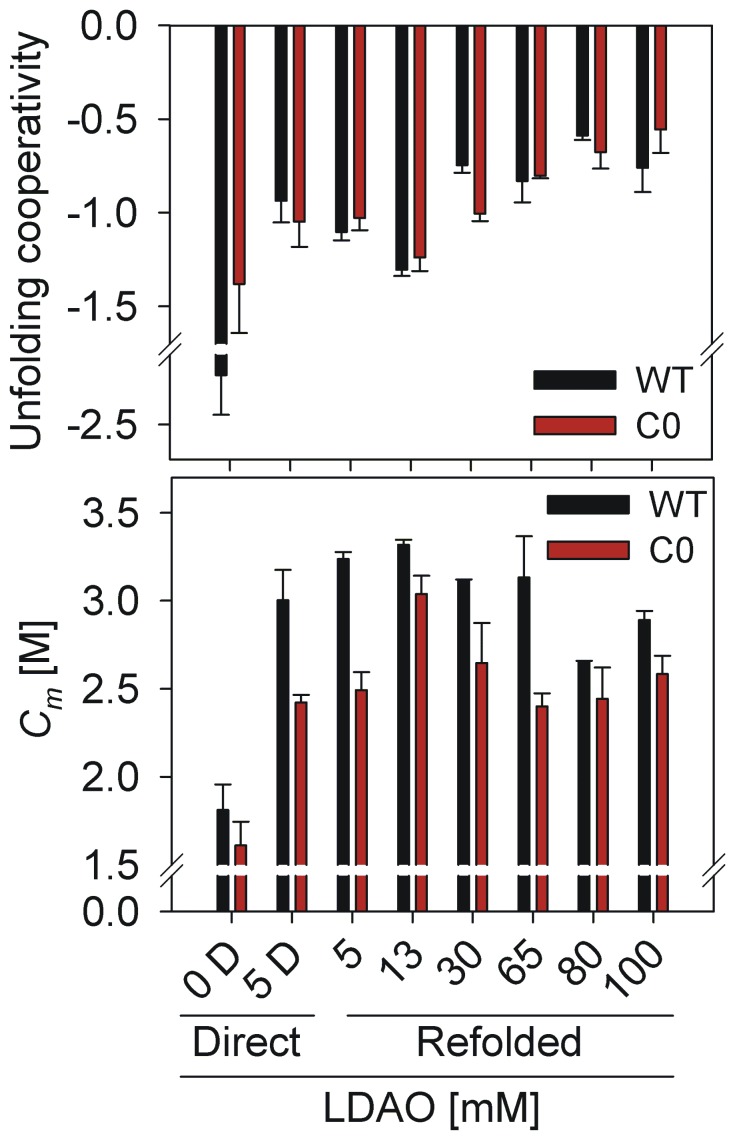
Thermodynamic parameters derived from equilibrium unfolding measurements of hVDAC-2 in LDAO micelles. (Top) Unfolding cooperativity, which corresponds to the slope of the unfolding transitions shown in Figure 6B, is compared for both WT (black) and C0 (red), in various LDAO concentrations. Note that the values are highest in 13 mM LDAO (corresponding to an LPR of 2600:1) for both refolded WT and C0, and are significantly lowered in high LDAO concentrations. (Bottom) *C_m_* values obtained from the fits shown in Figure 6B are compared for hVDAC-2 WT (black) and C0 (red). While *C_m_* values for hVDAC-2 WT are comparable between 5 – 65 mM LDAO, C0 exhibits an acute dependence on the LDAO concentration, and is most stable in 13 mM LDAO. Note that the parameters calculated for 80 mM and 100 mM LDAO were derived from linear extrapolation method, since we did not obtain defined pre- and post-transition baselines in these LDAO concentrations. In both the graphs, the sample without LDAO (protein in buffer) is denoted as ‘0 D’ and protein refolded directly in 5 mM LDAO, with LPR of 1000:1, is denoted ‘5 D’. All error bars indicate standard deviation for three independent experiments.

In attempts to derive the energy terms that define the barrel’s thermodynamic stability and the effect of LPR on these energies, we collected data on equilibrium unfolding of both WT and C0 using GdnHCl denaturation, by monitoring changes in the intrinsic Trp fluorescence.Change in the emission profile that is typically observed for hVDAC-2,upon protein unfolding, is shown in Figure 6A. Conformational equilibrium necessitates that the unfolding and refolding pathways are superposable, for the accurate determination of equilibrium free energy [27]. In such cases, the unfolding process follows a two-state transition from N↔U (native↔unfolded). However, while the unfolding process is reversible, we observed hysteresis in several LDAO concentrations used (data not shown), indicating that insurmountable energy barriers separate the N and U states. The occurrence of hysteresis preventsus from determining equilibrium free energy values for hVDAC-2 in LDAO within our experimental timeframes.Hence, we compared the*C_m_*for GdnHCl-mediated unfolding of both WT and C0 in the various LPRs. Unfolded fractions at various GdnHCl concentrations (Figure 6B),were fit to a two-state equation [41], and two parameters were considered: (i) unfolding *C_m_*(Figure 7 (bottom panel)) and (ii) unfolding cooperativity (Figure 7 (bottom panel)), given by the slope of theunfolding transition.

At LDAO concentrationsbetween 5 – 65 mM, the unfolding transition is cooperative, and we observe sigmoidal transition curves for both WT and C0 (Figure 6B). Furthermore, unfolding cooperativity is the highest for both proteins in 13 mM LDAO (LPR of 2600:1; Figure 7 (top panel)) and we observe a distinct broadening of the transition with concomitant loss in unfolding cooperativity with increase in LDAO (and LPR). At 80 mM and 100 mM LDAO, both WT and C0 lack defined pre- and post-transition baselines, and the unfolding is linearly proportional to increasing GdnHCl (Figure 6B).This could arise from lowering of the change in the accessible surface area, due to destabilization of the refolded protein even at zero denaturant, or incomplete protein unfolding in 6 M GdnHCl.However, the fluorescence lifetime and anisotropy values (Table 2) are similar for both WT and C0 in the various LPRs, suggesting that the barrel likely undergoes destabilization in high LDAO concentrations prior to the addition of any denaturant. While the unfolding cooperativity is similar for both WT and C0, a comparison of the *C_m_*values (Figure 7 (bottom panel))indicates a greater stability of the WT to chemical denaturation, in all lipid concentrations, while C0 exhibits highest *C_m_* values only at 13 mM LDAO.Particularly, C0 is substantially destabilized when the LDAO concentration is varied on either side of 13 mM (see Figure 7 (bottom panel)). On the other hand, WT exhibits comparable stability between LPRs of 2600∶1 to 13000∶1 and undergoes destabilization only in 80 mM and 100 mM LDAO. Our data suggests that mutation of cysteine residues of hVDAC-2 gives rise toan acute loss in LPR tolerance of the barrel.

Sincewe could not directlyestimate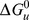, due to the occurrence of hysteresis in some LPRs, we compared the
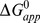
for both proteins 1 h after addition of denaturant, at 25°C (Table S1 in File S1). In line with the *C_m_* values, the data point to greater stability of WT over C0 by ∼0.7 – 1.0 kcal/mol. Both proteins display highest
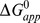
values for 13 mM LDAO thatcorrespond to ∼4.3 kcal/mol and ∼3.8 kcal/mol for WT and C0, respectively, reflecting the highest hVDAC-2 barrel stability in this LDAO concentration.It is also interesting to note that both
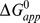
and *C_m­_* are lowered when the LPR is increased. Furthermore, we do not observe a distinct pattern in the change in 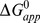 as a consequence of cysteine mutations. However, the overall values point to a marginally more stabilized WT protein in most LDAO concentrations, which is in good agreement with buried indole moieties of Trp residues for this protein (Figure 4), and the associated absence of complete barrel denaturation by urea in both LDAO and DDM (Figure 5).

## Discussion and Conclusion

Of the three human VDAC isoforms, hVDAC-2 and hVDAC-3 are enriched with cysteine residues (nine and six, respectively). hVDAC-1 cysteines are known to exist in the reduced state and are not required for barrel functioning [22]. We have previously demonstrated using mass spectrometry, that the cysteine residues of hVDAC-2, refolded in LDAO micelles, exist largely as free thiol moieties and are unlikely to form disulfide bonds *in vitro* [24]. Previous studies have proposed an alternative role for the cysteine residues in conferring localized quenching of reactive oxygen species generated in the mitochondrial inter-membrane space [60].In this study, we have probed the role of cysteines on the overall stability of the hVDAC-2 barrel and the dependence of this stability on LPR. Our study demonstrates that the unusual abundance of cysteines in hVDAC-2 influences the strength of interaction of the refolded barrel with its LDAO environment. For example, when we consider the lifetime and anisotropy measurements (Tables 2 and 3), it is apparent that the relative protein-micelle size and the degree of solvent exposure of tryptophan is similar for both the proteins. However, acrylamide has better access to the hVDAC-2 C0 tryptophans, giving rise to higher *K*_SV_ values. Accessibility of interfacial Trp residues could vary due to the extent of their association with the lipid or detergent environment used for protein folding.It is likely that in the hVDAC-2 C0 mutant, absence of Cys residues may lead to weakened barrel-LDAO interactions. We are tempted to speculate that this added structural role for cysteines also mediates efficient hVDAC-2 barrel interaction with its native lipid environment in the mitochondrial outer membrane.

Our study reveals that the presence of sufficient micellar forms (∼0.06 mM micelles in 5 mM ‘direct’ *vs* ∼0.85 mM micelles in 65 mM ‘refolded’ sample preparations) is critical for refolding hVDAC-2 *in vitro* (see Figure 2).LPR also plays a role in determining the hVDAC-2 barrel stability, with barrel destabilization observed in both high and low LPR conditions. The acute LPR dependence of the C0 mutant also allows us to conclude that the cysteine-enriched barrel displays a stronger interaction with its surrounding lipid milieu, when compared with a Cys-less VDAC. LDAO-to-protein ratio also plays a major role in hVDAC-2 kinetic stability, with LDAO concentrations in the range of 13 – 65 mM and LPRs of 2600∶1 – 13000:1emerging as key elements in both the folding and unfolding processes.Do these values bear implications on the *in vivo* regulation and recycling of VDAC isoforms with fewer cysteine residues?The biological relevance of these LPRs is presently unclear, since LDAO does not necessarily mimic the observed dielectric constant, elastic properties etc., of the native lipid bilayer surrounding hVDAC-2 in the mitochondrial outer membrane. Furthermore, our observations are in micellar systems, and they may not be directly translatable to barrel behavior *in vivo*. Previous studies suggest that membrane proteins show similar secondary and tertiary structures in detergent micelles as well as lipid bilayers [61]–[63], which leads us to speculate that lateral pressure generated by densely packed LDAO micellesmay possibly mimic that of lipid bilayers, and cause hVDAC-2 destabilization. This is further alleviated by the presence of salt, as it lowers electrostatic repulsion of the headgroups and promotes packing [64]. Rapid protein unfolding in high LDAO therefore ensues upon perturbation by chemical denaturants such as GdnHCl. The observed destabilization of hVDAC-2 in dimyristoyl phosphatidylcholine vesicles [24], strengthens our speculations.

**Figure 8:**
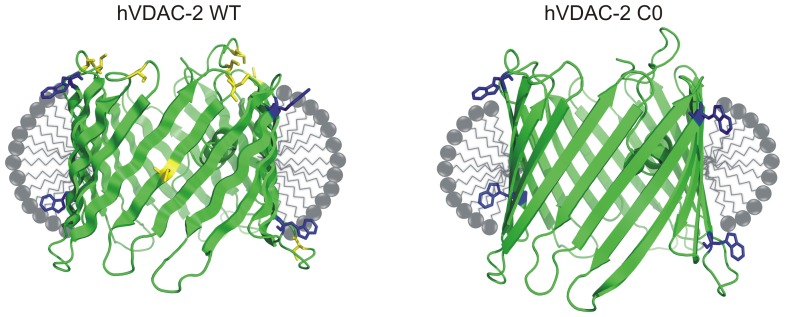
Schematic illustration of the relationship between hVDAC-2 stability and cysteine residues. The nine cysteine residues in hVDAC-2 WT stabilize the refolded barrel by strong protein-LDAO interactions. On the contrary, C0 shows poorer interaction efficiency with the surrounding LDAO milieu. Previous studies have indicated that the WT barrel shows less thermal stability compared to the Cys-less protein [24]. This indicates that hVDAC-2 WT possibly possesses weaker or fewer interstrand interactions and forms a ‘less structured’ barrel. On the other hand, the existence of strong intra-protein interactions in hVDAC-2 C0, evident from its greater thermal stability [24], contradicts the observed destabilization of this construct under equilibrium chemical unfolding. We speculate that native hVDAC-2 compensates for the poorer intra-protein interactions by the formation of strong association with its lipid or detergent environment, illustrated here as additional detergent molecules in the micelle (detergent molecules are shown in grey). The well-structured barrel in C0 retains fewer stabilizing points of association with LDAO, resulting in compromised barrel-LDAO interactions, which in turn leads to the observed destabilization of C0. The tryptophan and cysteine residues have been shown in blue and yellow, respectively.

Chemical denaturation measurements reveal that WT hVDAC-2 is more tolerant than C0 to chaotropic agents, possibly because of the strong barrel-lipid interactions. This is schematically represented in Figure 8.We speculate that the cysteine residues of human VDAC isoform 2 are key contributors for maintaining optimal interactions of this anion channel with its apolar environment. While this may result in a ‘noisier’ channel with conductance properties that are possibly less efficient than the more abundant isoform 1 (see Figure 1, and Figure S3 in File S1), it is interesting to speculate that the ‘less structured’ hVDAC-2 barrel which demonstrates strong(er) lipid interactions may be evolutionarily selected for its anti-apoptotic properties. Additionally, the occurrence of hysteresis in the (un)folding pathway indicates kinetic stability of the hVDAC-2 barrel, which may further be alleviated by barrel oligomerization *in vivo*. Whether hVDAC-2 employs oligomerization as an evolutionarily selected mechanism for transmembrane barrel stability is presently unknown [33]. Nevertheless, if hVDAC-2 oligomerization does occur *in vivo*, it is likely to be independent of the cysteines, and may not necessarily be induced by changes in the lipid environment.It must be noted that our experiments do not completely exclude disulfide bond formation *in vivo*, and it is possible that in the lipid bilayer, transient intra- and intermolecular disulfides do exist for hVDAC-2.

Hysteresis could also result as a consequence of the experimental set-up, as observed in hVDAC-2 denaturation using urea, wherein protein unfolding is largely prevented by LDAO micelles occluding the accessibility of urea to the barrel interior.Evidence supporting LDAO-urea interaction can be obtained from reports on the natural osmolyte TMAO. The LDAO headgroup is structurally similar to TMAO, which is known to counteract the action of urea on proteins (see [65], [66] and references therein). Although its mechanism is still debated, TMAO is hypothesized to sequester urea from the protein by formation of hydrogen bonds. We speculate that LDAO, being structurally similar to TMAO displays a similar behavior, impeding urea-mediated protein denaturation, and thereby increasing the protein *C_m_* beyond limits of experimental determination. Further confirmation for this phenomenon can be found in the studies on OmpA refolded in LDAO *vs* amphipol A8-35 [29]. Moreover, TMAO has been found to only partially perturb the ability of GdnHCl to denature peptides [65], explaining our observation with LDAO and GdnHCl (compare Figure 5 and Figure 6).

In conclusion, we observe that in high LDAO concentrations (and LPRs), barrel destabilization precedes the effect of a denaturant, and lowers unfolding cooperativity by destabilizing the native state, without loss in the secondary structure content. This could arise from the formation of stable LDAO-bound structured segments of the protein due to excessive micelles in the sample [67], which results in weaker intra-protein interactions.The *in vivo* protein content of the mitochondrial outer membrane is far less than the inner membrane, giving rise to high LPR [68], which should cause hVDAC-2 barrel destabilization. Native hVDAC-2 structure has therefore naturally evolved a certain degree of structural imperfection, so as to facilitate better barrel accommodation in, and interaction with, its lipid milieu. It is likely that the destabilization is counteracted by strong association with apoptotic factors including BAK [11], [69]–[71].Furthermore, it has not escaped our notice that oxidation of the free thiol moieties under oxidative stress conditions in the cell could give rise to an altered barrel structure which now possesses biophysical properties similar to our Cys-less mutant. A consequence of this subtle structural variation and lipid association affinity may be the triggering of cell death through non-apoptotic mechanisms.

## Supporting Information

File S1 (PDF)
