# Supplementary material for: Cysteine Residues Impact the Stability and Micelle Interaction Dynamics of the Human Mitochondrial β-Barrel Anion Channel hVDAC-2
Source: PLoS One. 2014 Mar 18;9(3):e92183. doi: 10.1371/journal.pone.0092183 (PMC3967697; doi:10.1371/journal.pone.0092183)
Supplement: File S1 — (PDF) [file pone.0092183.s001.pdf]

Cysteine Residues Impact the Stability and Micelle Interaction Dynamics of the Human Mitochondrial β-barrel Anion Channel hVDAC-2

Svetlana Rajkumar Maurya and Radhakrishnan Mahalakshmi*

Supplemental Information

SUPPLEMENTAL FIGURES

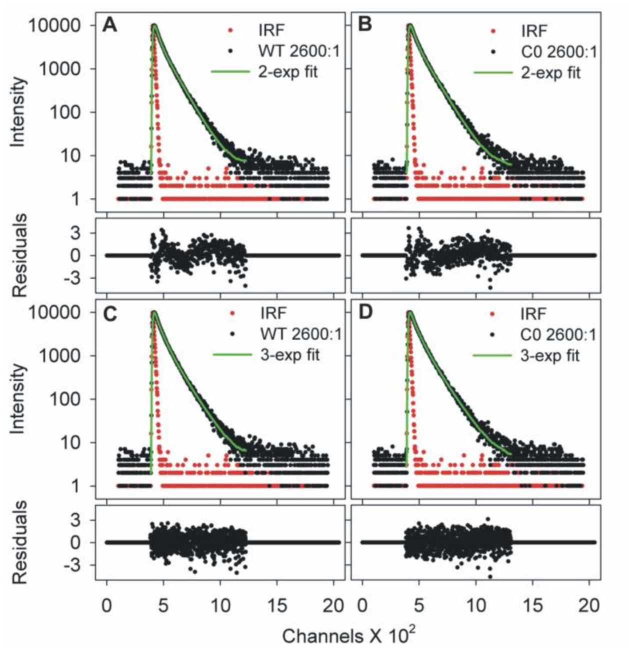

Figure S1. Analyses of tryptophan fluorescence lifetimes. Sample graphs highlighting the difference in confidence of fits between double and triple exponential functions for WT (left panel) and C0 (right panel) refolded in LPR of 2600:1. Triple exponential fits (3-exp fit; C and D) provided better residuals and χ^2 values in comparison with the double exponential fits (2-exp fit; A and B), in line with observations for soluble proteins [1].

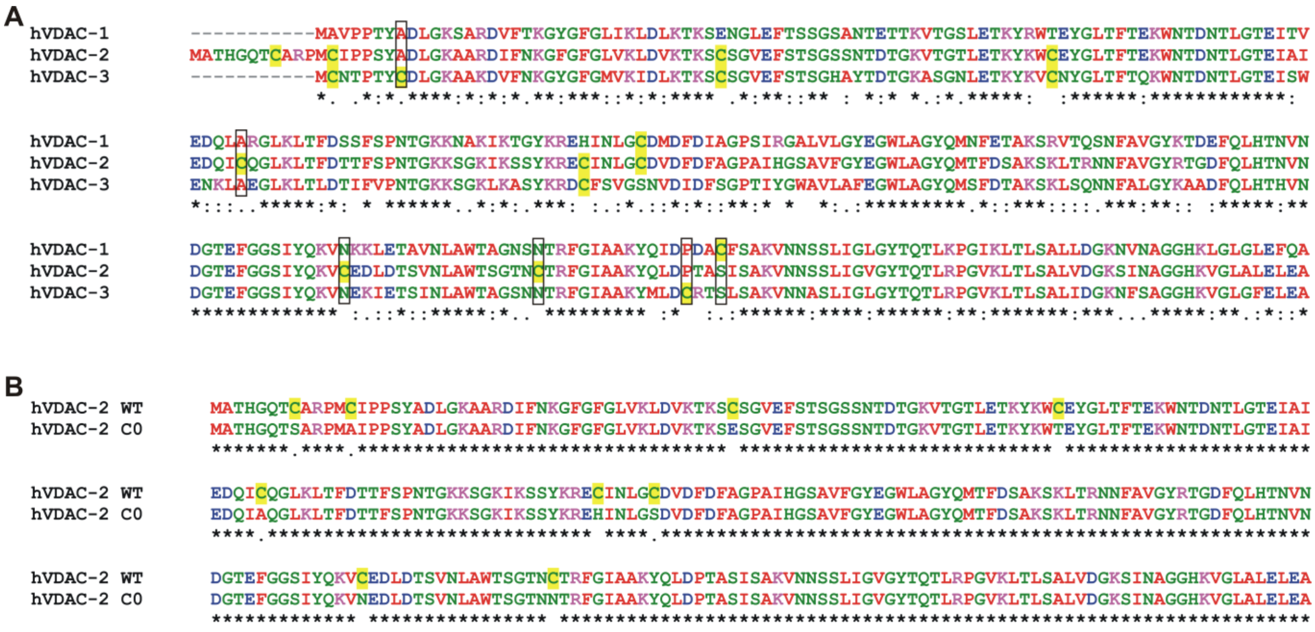

Figure S2. Multiple sequence alignment (MSA) of human VDAC isoforms and mutants. (A) The human genome has three known isoforms of VDAC, namely hVDAC-1, 2 and 3. The sequence alignment demonstrates that the three isoforms share ~70% sequence identity to each other. hVDAC-2 cysteines having a counterpart in either hVDAC-1 or 3 have been boxed. These residues were mutated to the corresponding amino acids found in hVDAC-1 or hVDAC-3, to generate the Cys-less hVDAC-2 mutant C0. Cys8 is unique to hVDAC-2 and lacks a counterpart in the other two isoforms; this was mutated to Ser (C8S). (B) MSA of hVDAC-2 WT and C0 highlighting the differences between the two mutants. In both MSAs, the cysteine residues are highlighted in yellow and the MSA have been generated using Clustal Omega [2]).

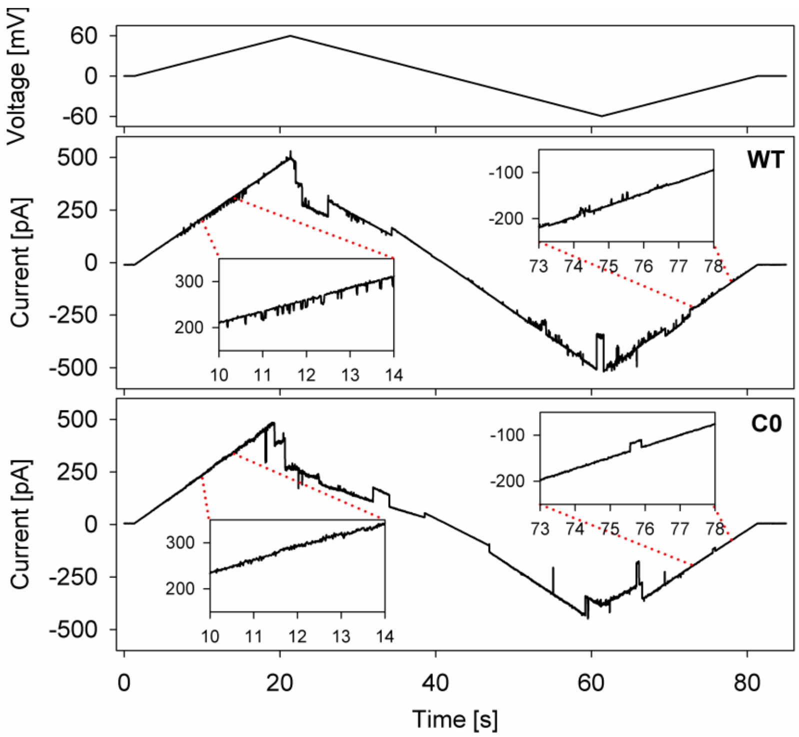

Figure S3. Channel conductance measurements of hVDAC-2 WT and C0 against a voltage ramp (Continued from Figure 1). Representative results of voltage ramp experiments comparing the channel conductance of full-length hVDAC-2 WT and C0 protein. A voltage ramp ranging from +60 mV to -60 mV at a rate of 3 mV/s was applied, and is shown in the upper panel. The change in the conductance of hVDAC-2 WT (middle panel) and C0 (lower panel) in response to the voltage are shown. In both the cases presented here, the membrane had ~3-4 active channels. Insets show expansions of channel conductance to highlight noisier channels in the case of WT.

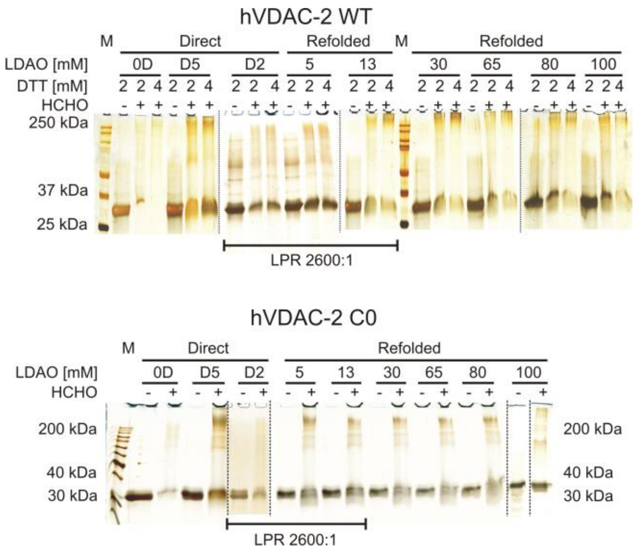

Figure S4. Formaldehyde cross linking of hVDAC-2 WT and C0 at various LDAO concentrations. Refolded samples in different LDAO concentrations were subjected to cross linking in the presence of 2% formaldehyde (HCHO; '–' and '+' indicate absence and presence of formaldehyde in the reaction), and unboiled samples run on a 12% SDS-PAGE were visualized by silver staining. Shown here are representative gels for both proteins in the various LPRs described in this study. As controls, samples directly refolded in LPR of 0:1 (0 mM LDAO, 0D; unfolded control), 1000:1 (5 mM LDAO, D5) and 2600:1 (5 mM LDAO, D2) (D2 and D5 are labeled as 'Direct') were also cross linked. These samples show a greater content of oligomeric species, likely arising from aggregation. Both refolded WT and C0, prepared by dilution of the refolded stock, showed comparable levels of oligomeric species (~40%) in all the LPRs examined; the results therefore suggest that LDAO has no overwhelming contribution to barrel multimerization. The lane containing the protein molecular weight standards is marked as 'M' and relevant molecular weights are indicated. Dashed lines demarcate different gels that are presented together for comparison.

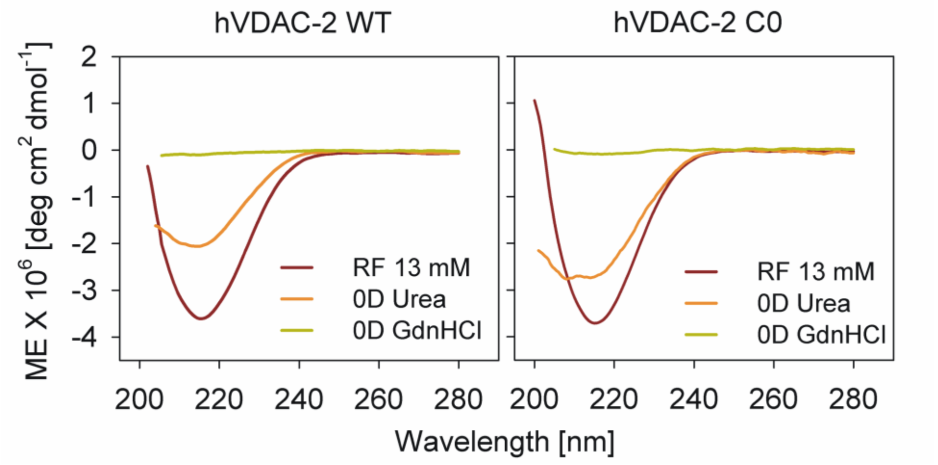

Figure S5. CD wavelength scans of aggregated proteins. Far-UV CD scans of aggregated proteins prepared by 50-fold dilution of protein stock in 8 M urea (0D Urea) and 6 M GdnHCl (0D GdnHCl) into 50 mM phosphate buffer pH 7.2 and 100 mM NaCl. The ‘0D Urea’ sample did not show visible precipitation. However, we observe moderate CD spectrum with a shallow trough centered at ~215 nm, corresponding to an extended conformation. Molar ellipticity (ME) values of the refolded protein in LDAO with LPR of 2600:1 (RF 13 mM) is shown here for comparison, to highlight the increase in secondary structure content upon addition of a detergent. We observed extensive precipitation in the case of ‘0D GdnHCl’ sample. The observed ME values are very low in this condition, which could arise from the lack of defined secondary structure in this protein or the extensive scattering of light by the particulate material. Nevertheless, the precedence of aggregation suggests that hVDAC-2 diluted into buffer from GdnHCl does not adopt sufficient local structure to bury the hydrophobic groups distributed in the protein.

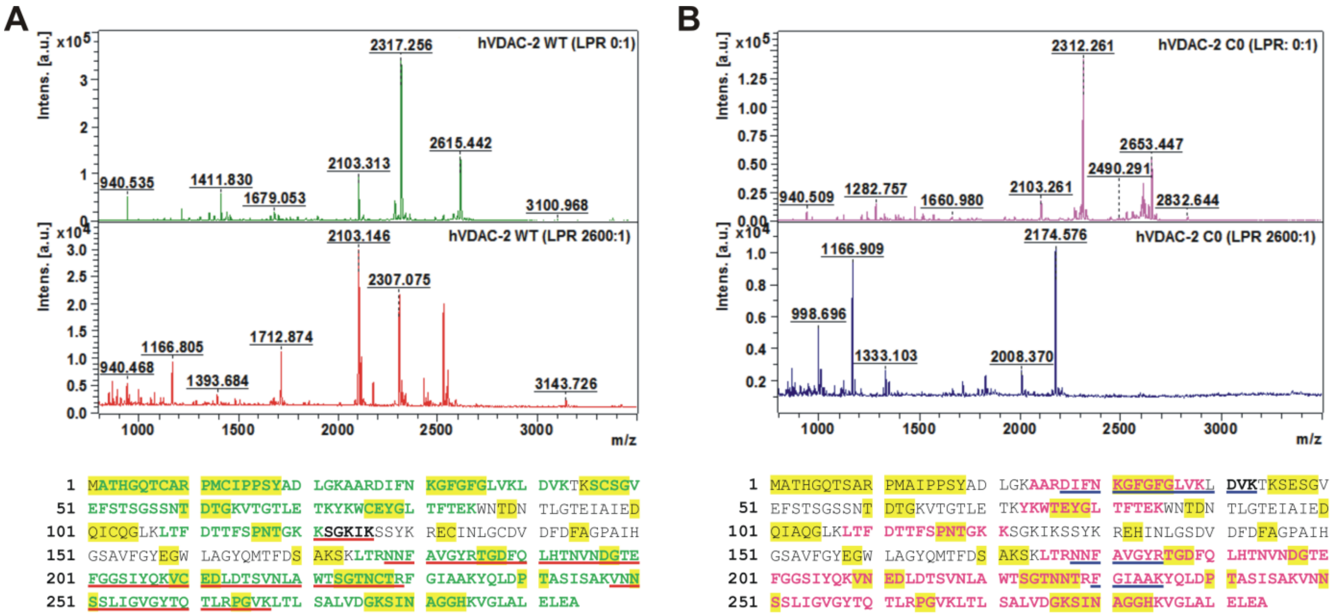

Figure S6. Mass spectrometric analysis of tryptic digests of aggregated and refolded hVDAC-2 protein. hVDAC-2 WT (A) and C0 (B) proteins, in an LPR of 0:1 (aggregated protein without LDAO, green and pink respectively) and 2600:1 (refolded in LDAO, red and blue respectively), were subjected to in-solution trypsin digestion for 10 min at 25 °C. The reaction was arrested by the addition of 0.1% TFA and subjected to peptide mass fingerprinting on a MALDI-ToF/ToF mass spectrometer, using established protocols [3]. Aggregated samples provided clean spectra (top spectra in both A and B), whereas we observed difficulties in obtaining good S/N in LDAO-containing refolded samples (bottom spectra in both A and B), due to interference from the detergent. In the samples with LPR 0:1 (aggregated proteins), peptide fragments which could be mapped to the protein sequence (shown below the spectra) are indicated in bold and color coded to match the corresponding samples (WT, green; C0, pink). Peaks obtained in the refolded protein samples (LPR 2600:1) that mapped to corresponding regions in the protein sequence are underlined, and are color coded (WT, red; C0, blue). Regions which correspond to the loops in the I-TASSER [4] modeled structure of hVDAC-2 have been highlighted in yellow in both the sequences (WT and C0). Note that in the LPR 0:1 samples, >73 % sequence coverage is obtained in the case of WT. In the refolded proteins, the few peaks that are indeed observed map to similar regions of the aggregated samples. However, due to the limited number of peaks observed in the refolded protein (<27 % sequence coverage for WT), it is difficult to draw a direct correlation of the trypsin-accessible sites in the refolded and aggregated samples, as observed from SDS-PAGE (Figure 3). Nevertheless, we anticipate a similar peptide mass fingerprint for both the refolded and aggregated proteins, as the R/K-rich regions would be solvent exposed and trypsin accessible in both protein forms.

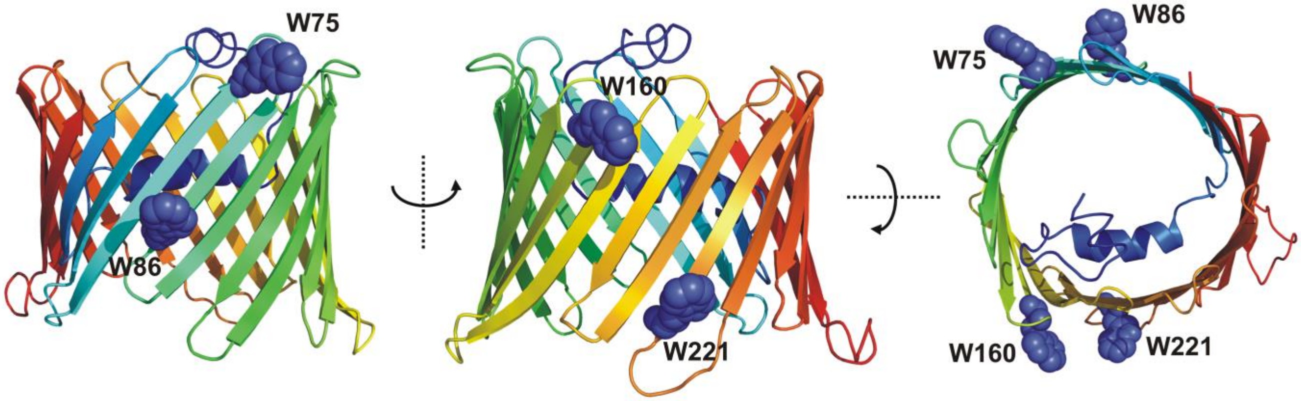

Figure S7. Ribbon diagram of hVDAC-2 highlighting the indole ring orientation of the four Trp residues. hVDAC-2 WT structure was modeled using the crystal (2JK4; [5]) and NMR (2K4T; [6]) structures of hVDAC-1 as templates, on I-TASSER [4]. Side chains of the four Trp residues are represented as blue spheres, and are expected to be positioned at the solvent-lipid interface in the refolded 19-stranded barrel model.

TABLES

Table S1. Thermodynamic parameters obtained from the GdnHCl equilibrium unfolding of refolded hVDAC-2

LDAO [mM]	hVDAC-2 WT			hVDAC-2 C0			$\Delta\Delta G^0_{app}$ [b]
	Unfolding cooperativity [a]	C_m [M]	ΔG^0_{app} [b]	Unfolding cooperativity [a]	C_m [M]	ΔG^0_{app} [b]	
5 [c]	-1.10 ± 0.05	3.24 ± 0.04	3.57 ± 0.10	-1.03 ± 0.07	2.49 ± 0.10	2.57 ± 0.27	1.00
13 [c]	-1.30 ± 0.04	3.32 ± 0.03	4.33 ± 0.08	-1.24 ± 0.07	3.04 ± 0.10	3.76 ± 0.09	0.57
30 [c]	-0.75 ± 0.04	3.12 ± 0.002	2.33 ± 0.13	-1.01 ± 0.04	2.65 ± 0.23	2.66 ± 0.12	-0.33
65 [c]	-0.83 ± 0.11	3.13 ± 0.23	2.58 ± 0.15	-0.80 ± 0.01	2.40 ± 0.07	1.93 ± 0.03	0.65
80 [d]	-0.59 ± 0.02	2.65 ± 0.007	1.56 ± 0.07	-0.68 ± 0.09	2.44 ± 0.18	1.64 ± 0.10	-0.08
100 [d]	-0.76 ± 0.13	2.89 ± 0.05	2.19 ± 0.34	-0.56 ± 0.13	2.58 ± 0.10	1.45 ± 0.39	0.74

[a] Units: kcal mol^{-1} M^{-1}; [b] Units: kcal mol^{-1}; [c] Derived from fits of the equilibrium unfolding curves to a two-state equation. These values are in good agreement with data derived from the linear extrapolation method; [d] Derived from linear extrapolation, due to lack of proper pre- and post- transition baselines.

References

1. Sarkar SS, Udgaonkar JB, Krishnamoorthy G (2011) Reduced fluorescence lifetime heterogeneity of 5-fluorotryptophan in comparison to tryptophan in proteins: implication for resonance energy transfer experiments. J Phys Chem B 115: 7479-7486.
2. Sievers F, Wilm A, Dineen D, Gibson TJ, Karplus K, et al. (2011) Fast, scalable generation of high-quality protein multiple sequence alignments using Clustal Omega. Mol Syst Biol 7: 539.
3. Maurya SR, Mahalakshmi R (2013) Modulation of Human Mitochondrial Voltage-dependent Anion Channel 2 (hVDAC-2) Structural Stability by Cysteine-assisted Barrel-lipid Interactions. J Biol Chem 288: 25584-25592.
4. Roy A, Kucukural A, Zhang Y (2010) I-TASSER: a unified platform for automated protein structure and function prediction. Nat Protoc 5: 725-738.
5. Bayrhuber M, Meins T, Habeck M, Becker S, Giller K, et al. (2008) Structure of the human voltage-dependent anion channel. Proc Natl Acad Sci U S A 105: 15370-15375.
6. Hiller S, Garces RG, Malia TJ, Orekhov VY, Colombini M, et al. (2008) Solution structure of the integral human membrane protein VDAC-1 in detergent micelles. Science 321: 1206-1210.
